# Computational Clues of Immunogenic Hotspots in *Plasmodium falciparum* Erythrocytic Stage Vaccine Candidate Antigens: In Silico Approach

**DOI:** 10.1155/2022/5886687

**Published:** 2022-10-13

**Authors:** Mojtaba Azimi-Resketi, Saeed Heydaryan, Niloufar Kumar, Azin Takalou, Reza Esmaeelzadeh Dizaji, Bahman Noroozi Gorgani, Morteza Shams

**Affiliations:** ^1^Department of Parasitology and Mycology, School of Medicine, Isfahan University of Medical Sciences, Isfahan, Iran; ^2^Department of Internal Medicine, Faculty of Veterinary Medicine, University of Tehran, Tehran, Iran; ^3^Department of Microbiology, Faculty of Veterinary Sciences, Science and Research Branch, Islamic Azad University, Tehran, Iran; ^4^Department of Microbiology and Immunology, Faculty of Veterinary Medicine, University of Tehran, Tehran, Iran; ^5^Doctorate in Veterinary Medicine, Sanandaj Branch, Islamic Azad University, Sanandaj, Iran; ^6^Zoonotic Diseases Research Center, Ilam University of Medical Sciences, Ilam, Iran

## Abstract

Malaria is the most pernicious parasitic infection, and *Plasmodium falciparum* is the most virulent species with substantial morbidity and mortality worldwide. The present in silico investigation was performed to reveal the biophysical characteristics and immunogenic epitopes of the 14 blood-stage proteins of the *P. falciparum* using comprehensive immunoinformatics approaches. For this aim, various web servers were employed to predict subcellular localization, antigenicity, allergenicity, solubility, physicochemical properties, posttranslational modification sites (PTMs), the presence of signal peptide, and transmembrane domains. Moreover, structural analysis for secondary and 3D model predictions were performed for all and stable proteins, respectively. Finally, human helper T lymphocyte (HTL) epitopes were predicted using HLA reference set of IEDB server and screened in terms of antigenicity, allergenicity, and IFN-*γ* induction as well as population coverage. Also, a multiserver B-cell epitope prediction was done with subsequent screening for antigenicity, allergenicity, and solubility. Altogether, these proteins showed appropriate antigenicity, abundant PTMs, and many B-cell and HTL epitopes, which could be directed for future vaccination studies in the context of multiepitope vaccine design.

## 1. Introduction

Human malaria disease has been a lethal parasitic infection during centuries and continues as one of the most prevalent infectious diseases with almost 228 million cases and 405000 deaths globally, according to the World Health Organization (WHO) [1, 2]. Five *Plasmodium* species, transmitted *via* bites of female *Anopheles* mosquitoes, are responsible for malaria outbreaks across the world, comprising the deadliest *Plasmodium falciparum* (*P. falciparum*, Pf), followed by *P. vivax*, *P. ovale*, *P. malariae*, and *P. knowelsi* [3]. Reportedly, Pf has been documented as the causative agent of 99.7% and 62.8% of human cases in tropical Africa and Southeast Asia, respectively [4, 5]. About 10-15 days postinoculation, symptoms of a mild illness would emerge, primarily manifested by head and body aches, chill, nausea, vomiting, and fever, whereas severe malaria cases suffer from multiorgan involvement in adults as well as respiratory distress and anemia in children [6].

There are multiple therapeutic agents with varying degrees of efficacy used to combat malaria; for instance, artemisinin-based combination therapies (ACTs), including artemisinin derivative plus antimalarial drugs such as mefloquine or lumefantrine, as well as durable injection of artesunate are administered to control uncomplicated and complicated malaria, respectively [7–9]. In addition to the drug resistance against most administered therapeutics, based on WHO reports, cross-resistance is a more complicated issue, which may be observed in case of those drugs with similar action mode or of the same chemical family [10]. Hence, a successful malaria elimination program requires the implementation of prophylactic along with therapeutic approaches. Thus far, there is no licensed human malaria vaccine, while such an advantageous vaccine has been targeted by WHO to be available by 2030s [11]. Currently, RTS, S/AS01 based on segments of the circumsporozoite (CSP) protein of the African strain 3D7, is a well-known vaccine candidate which passed phase III trials in African countries [12].

The vaccine production strategies against malaria parasites is commonly based on their complicated life cycle, being sorted into three categories: (i) preerythrocytic vaccines which target sporozoites entering liver cells [13]; (ii) erythrocytic vaccines, targeting asexual stages within red blood cells (RBCs) [14]; and (iii) transmission-blocking vaccines, targeting sexual stages in the mosquito gut [15]. Because blood-stage infection is responsible for major malaria-induced pathology and clinical disease, there exist a robust rationale for the development of erythrocytic stage vaccines [16]. At this stage, merozoites successively invade and replicate within erythrocytes and cause cell burst ([17]); hence, they are easily exposed to the host's humoral immune responses, which in turn facilitate the control of parasitemia or even a partial protection [18, 19]. On this basis, blood-stage antigens are of utmost interest in seeking an appropriate vaccine candidate and have been shown to be protective, to some extent, in laboratory animal models [20–23] and in humans [24]. Also, simultaneous targeting of an array of antigens would demonstrate a more potent inhibitory effects on Pf [25, 26]. Based on previous studies, several blood-stage vaccine candidate antigens have been discovered in *P. falciparum*, including apical membrane antigen 1 (AMA1); rhoptry neck 2 (Ron2); erythrocyte-binding antigen 175 (Eba175); reticulocyte-binding protein homolog 5 (PfRh5); merozoite Rh5 interacting protein; cysteine-rich protective antigen (CyRPA); merozoite surface proteins (MSP) 1, 3, 4, and 6; the rhoptry-associated, leucine zipper-like protein 1 (Ralp1); ring-infected erythrocyte surface antigen (RESA); and serine repeat antigens (Sera) 5 and 8 [27].

Advances in the vaccinology studies during last decades have promoted our knowledge on the novel vaccine development methods [28]. Subunit-based vaccines are safe and efficient procedures in vaccine researches, based on the selection and incorporation of different epitope and/or antigens [29]. In this sense, unprecedented computational tools facilitate the identification and screening of potent vaccine candidates and/or their immunogenic epitopes within a given organism. Such approaches can target specific immune responses in a time- and cost-effective manner [30]. The present study aimed at the prediction of some basic features (antigenicity, allergenicity, physicochemical properties, posttranslational modification (PTM) sites, signal peptide, transmembrane domains, subcellular localization, and structural analysis) and structural analysis of the aforementioned 14 erythrocytic stage Pf antigens along with the prediction of the potent B-cell and helper T lymphocyte (HTL) epitopes through comprehensive immunoinformatics approaches.

## 2. Methods

### 2.1. Retrieval of Amino Acid Sequences of 14 Blood-Stage Proteins of Pf

Amino acid sequences of the selected blood-stage antigens of Pf were retrieved through the freely accessible UniProt Knowledgebase (UniProt KB) (https://www.uniprot.org/) [31], as follows: AMA1 (P22621), CyRPA (Q8IFM8), Eba175 (P19214), MSP1 (P04933), MSP3 (A0A7D5SLD3), MSP4 (O76244), MSP6 (C6ZGD4), Ralp1 (A0A193PDF2), RESA (P13830), Rh5 (Q8IFM5), Ripr (O97302), Ron2 (B9A598), Sera5 (Q9TY95), and Sera8 (A0A0E3VL24).

### 2.2. Prediction of Basic Biochemical Characteristics of Erythrocytic Stage Antigens

Some of the fundamental biochemical properties of the selected proteins were predicted using different web servers. For antigenicity, VaxiJen v2.0 (threshold: 0.45) possesses 78.0% prediction accuracy, based on physicochemical features of a protein (http://www.ddg-pharmfac.net/vaxijen/VaxiJen/VaxiJen.html) [32]. Also, ANTIGENpro, available at http://scratch.proteomics.ics.uci.edu/, is an alignment- and pathogen-free predictor of antigenicity using microarray data. Another important aspect of a vaccine candidate is lack of allergenicity, which was predicted using two web servers [33]. AllergenFP v1.0, available at https://ddg-pharmfac.net/AllergenFP/method.html, differentiates allergens from nonallergens with a descriptor fingerprint approach [34], whereas AllerTOP v2.0, available at https://www.ddg-pharmfac.net/AllerTOP/method.html, performs mostly through *k*-nearest neighbors (k NN) [35]. The solubility of each protein was predicted using Protein-Sol server (https://protein-sol.manchester.ac.uk/), based on available data for *Escherichia coli* protein solubility in a cell-free expression system [36].

Preliminary physicochemical properties of the erythrocytic stage proteins were predicted using ProtParam server, available at https://web.expasy.org/protparam/, which estimates the molecular weight (MW), positively and negatively charged residues, isoelectric point (pI), *in vitro* and *in vivo* estimated half-life, instability index, aliphatic index, and grand average of hydropathicity (GRAVY) [37]. Moreover, three online bioinformatics tools from DTU Health Tech Services server (Denmark), available at https://services.healthtech.dtu.dk/, including SignalP-6.0 [38], Deep TMHMM [39], and DeepLoc-2.0 [40] were used to characterize the putative signal peptide, transmembrane domain, and subcellular localization for each protein.

### 2.3. Prediction of Major Posttranslational Modification (PTM) Sites

A number of PTM sites were predicted for *P. falciparum* blood-stage proteins, including phosphorylation, palmitoylation, lysine acetylation, O-glycosylation, and N-glycosylation. For this purpose, online tools of the DTU Health Tech Services (Denmark) including NetPhos 3.1, NetOGlyc 4.0, and NetNGlyc 1.0 (https://services.healthtech.dtu.dk/) as well as tools of the Cuckoo workgroup (http://biocuckoo.org/) such as CSS-Palm and GPS-Pail 2.0 were employed. “All Asn residues” option was used for NetNGlyc 1.0 prediction, while default parameters were applied to NetOGlyc 4.0 server.

### 2.4. Secondary and Three-Dimensional (3D) Model Prediction

To predict the secondary structures of each examined protein of Pf, amino acid sequences were submitted to the GOR IV web server, available at https://npsa-prabi.ibcp.fr/cgi-bin/npsa_automat.pl?page=/NPSA/npsa_gor4.html. This server predicts three main secondary structures, including alpha helix, extended strands, and random coils, within a given sequence [41]. In the following, the fully automated protein homology modeling server, SWISS-MODEL, was used to predict the 3D structure of stable proteins [42], including AMA1, CyRPA, MSP1, Rh5, and Sera5.

### 2.5. Prediction of Continuous and Conformational B-Cell Epitopes

A multistep approach was employed regarding prediction of 14-mer linear B-cell epitopes; hence, three web servers, ABCpred (http://crdd.osdd.net/raghava/abcpred/) with 0.8 threshold [43], BCPRED (http://ailab-projects1.ist.psu.edu:8080/bcpred/) with 80% threshold [44], and BepiPred (https://services.healthtech.dtu.dk/service.php?BepiPred-2.0) [45], were used, based on different machine learning methods. Shared epitopes in each protein sequence were then spotted and selected for further screening in terms of antigenicity, allergenicity, and water solubility using VaxiJen, AllergenFP, and PepCalc (https://pepcalc.com/) [46] web servers, respectively. Also, conformational B-cell epitopes of those stable proteins were predicted using ElliPro online tool, available at http://tools.iedb.org/ellipro/.

### 2.6. Prediction and Screening of Human Helper T Lymphocyte (HTL) Epitopes with Population Coverage Analysis

For this aim, major histocompatibility complex (MHC)-II binding tool of the Immune Epitope Database (IEDB) server was selected and human HTL epitope prediction was done using recommended method 2.22 and selecting “full human leukocyte antigen (HLA) reference set” option to predict 15-mer epitopes. Potential binders had a lower percentile rank in the provided results table. In the following, the antigenicity, allergenicity, and IFN-*γ*-inducing capacity of each epitope was estimated using VaxiJen, AllerTOP, and IFNepitope (http://crdd.osdd.net/raghava/ifnepitope/) online tools, respectively. The population coverage analysis was, also, done by the Population Coverage tool in IEDB server using all predicted epitopes.

### 2.7. Immune Simulation

The immune responses provoked by the five stable erythrocytic stage proteins (AMA1, CyRPA, Rh5, MSP1, and Sera5) were predicted in silico, using C-ImmSim web server, available at http://150.146.2.1/C-IMMSIM/index.php. This virtual simulation process was accomplished using default parameters with random seed 12345, simulation volume 10, and simulation steps 100. A PSSM for ML methods are the basis for the predictions in this server. The output indicates to three stimulated regions including the bone marrow, thymus, and lymph node [47, 48].

## 3. Results

**Figure 1:**
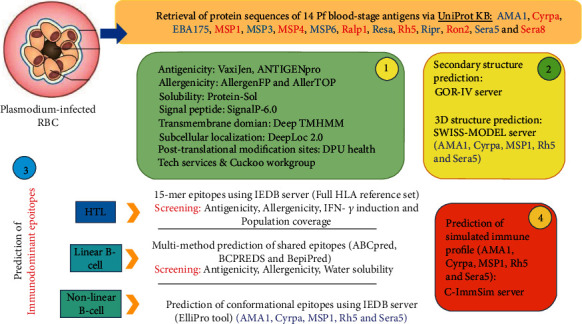
Schematic representation of the whole study design, prediction web servers, and screening methods regarding 14 Pf blood-stage vaccine candidate antigens [49].

A schematic representation of the whole study design is illustrated in Figure 1.

### 3.1. Antigenicity, Allergenicity, Solubility, and Other Physicochemical Characteristics

With respect to antigenicity prediction by VaxiJen server, MSP4 possessed highest score (1.0076), followed by MSP3 (0.8309), Sera5 (0.7150), and Eba175 (0.6903). Of note, Rh5 and Ripr showed least antigenic indices as 0.4882 and 0.4980, respectively. Using AllergenFP and AllerTOP servers, 3 (RESA, Sera5, and Sera8) and 5 proteins (AMA1, CyRPA, MSP3, RESA and Ripr) were found to be allergenic in nature. Based on Protein-Sol server, AMA1, Ripr, Ron2, and Sera8 showed to be low solubility scores, below threshold (0.45), whereas MSP3 and MSP4 were demonstrated to be highly soluble (0.806 vs. 0.894). Among blood-stage antigens of Pf, MSP1 had the longest protein sequence and heaviest MW with 1639 residues and ~187 kDa, while MSP4 had the smallest amino acid sequence and MW with only 272 residues and ~30 kDa, respectively. The pI of the examined proteins ranged from 4.46 in RESA to 9.37 in Ron2. In total, the predicted number of negatively charged residues was higher in all proteins, with the exception of Rh5, Ron2, and Sera8. The estimated half-life was 30 hours in mammalian reticulocytes for all proteins, except of Ralp1, which was predicted to be 1.1 hours. Out of 14 submitted proteins, 6 were shown to be stable, including AMA1, CyRPA, MSP1, Rh5, Sera5, and Sera8, while others were unstable. No dramatic variations were found regarding aliphatic index among examined proteins, and the highest and lowest scores belonged to CyRPA (88.23) and MSP3 (57.82), respectively. Moreover, a negative GRAVY score was estimated for all proteins, indicating hydrophilic trait in nature (Table 1).

### 3.2. Prediction of Signal Peptide, Transmembrane Domain, and Subcellular Localization

According to the SignalP server output, 8 proteins were demonstrated to possess signal peptide, comprising AMA1, CyRPA, Eba175, MSP1, MSP3, Ripr, Sera5, and Sera8. Based on deep TMHMM server, two proteins (AMA1 and RESA) had potential transmembrane domains. Furthermore, the subcellular localization of each protein was estimated and predicted using DeepLoc online tool. Full details of the results are provided in Table 1.

### 3.3. Prediction of Potential PTM Sites

Based on NetPhos online tool, MSP1, Eba175, and Ron2 proteins showed abundant phosphorylation sites in their sequences, while lower phosphorylation regions were detected in MSP4 and CyRPA. Palmitoylation sites were predicted in all proteins, except for MSP3, MSP6, and Ron2. Also, the highest number of N- and O-glycosylation sites were found in Eba175 protein. Lysine acetylation was, also, a prominent feature in MSP3 and Ron2 proteins (Table 2).

### 3.4. Structural Evaluation of Blood-Stage Proteins

**Figure 2:**
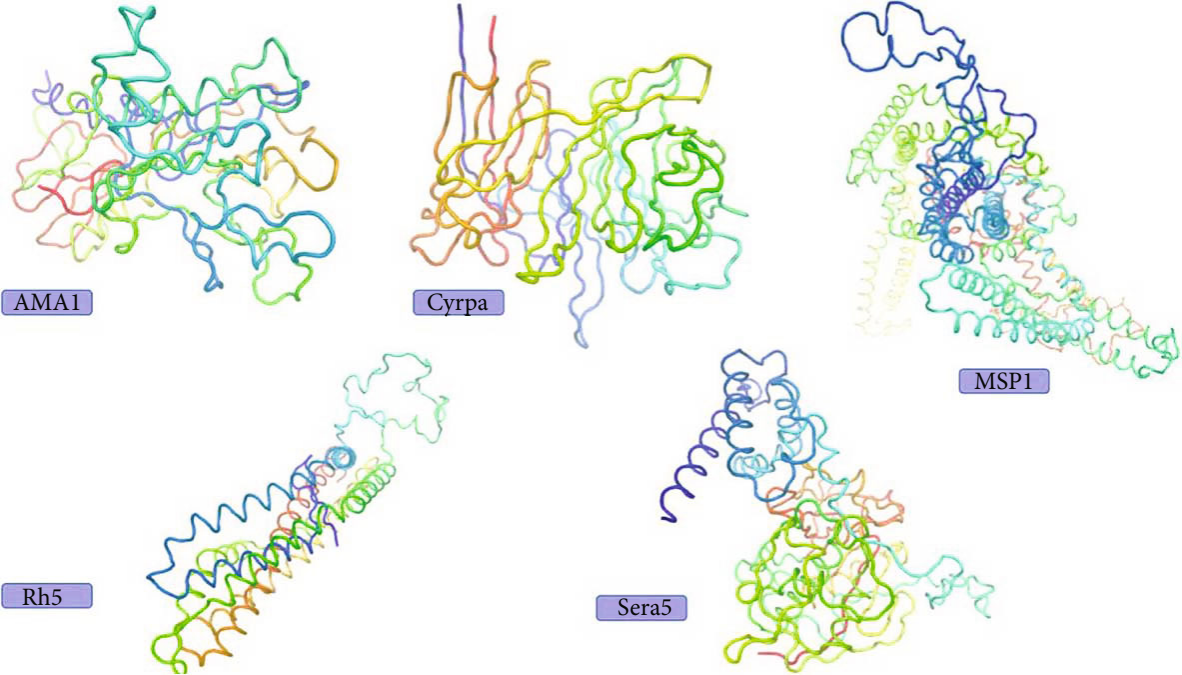
3D structure of five stable Pf blood-stage vaccine candidates (AMA1, CyRPA, MSP1, Rh5, and Sera5), based on SWISS-MODEL server predictions.

Secondary structure of the blood-stage Pf proteins was evaluated using GOR IV web server. Based on the server output, random coils were the most prominent secondary structure found in 10 proteins (AMA1, CyRPA, Eba175, MSP4, MSP6, Ralp1, Rh5, Ripr, Sera5, and Sera8), while in 4 proteins (MSP1, MSP3, RESA, and Ron2) alpha helix structures were prevalent.  Table 3 demonstrates the number and percentage of each predicted secondary structure within submitted Pf proteins. The 3D model of five stable proteins was predicted and illustrated using SWISS-MODEL web server, derived from templates 4r19.2.A, 7pi2.2.A, 6zbj.1.A, 4u0r.1.A, and 6x44.1.A, respectively (Figure). Of note, the sequence identity for above proteins was 99.70%, 99.10%, 62.52%, 99.61%, and 100%, respectively (Figure 2).

### 3.5. Prediction of Linear and Conformational B-Cell Epitopes

**Figure 3:**
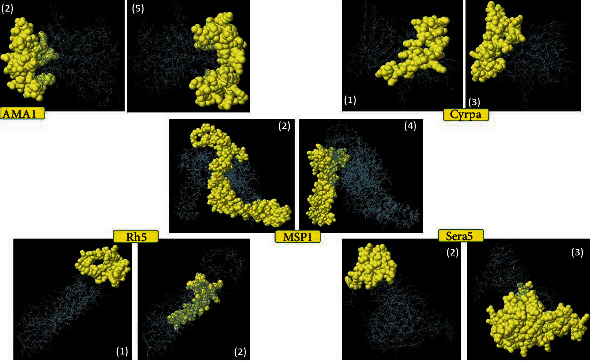
Illustration of highly populated and top-scored conformational B-cell epitopes in five modeled Pf blood-stage vaccine candidates (AMA1, CyRPA, MSP1, Rh5, and Sera5).

A robust multistep modality was designed and exerted to predict and screen potential B-cell epitopes in the selected Pf proteins. For this purpose, outputs of three web tools, including ABCpred, BCPRED, and BepiPred, were compared with each other and common epitopic regions were spotted and selected for further screening in terms of antigenicity, allergenicity, and water solubility. Our results showed 60 strictly chosen linear B-cell epitopes from 14 Pf blood-stage vaccine candidates which were highly antigenic and nonallergenic with good water solubility, as presented in Table 4. Moreover, conformational B-cell epitopes predicted for modeled protein structures (AMA1, CyRPA, MSP1, Rh5, and Sera5) are provided in Table 5. Of note, those highly populated noncontinuous B-cell epitopes with highest scores were illustrated (Figure 3).

### 3.6. Prediction, Screening, and Population Coverage Analysis of the Human HTL Epitopes

Based on IEDB HLA reference set, top five HTL epitopes for each submitted protein sequence were predicted and evaluated regarding antigenicity, allergenicity, and IFN-*γ* induction. Our results demonstrated that 24 epitopes were estimated to be potent IFN-*γ* inducers, including “GRPHIFAYVDVEEII” (CyRPA); “CNISIYFFASFFVLY, ISIYFFASFFVLYFA, KCNISIYFFASFFVL, and NISIYFFASFFVLYF” (Eba175); “HLYIYINNVASKEIV, LYIYINNVASKEIVK, and YIYINNVASKEIVKK” (MSP3); “CVELLSLASSSLNLI, ECVELLSLASSSLNL, VELLSLASSSLNLIF, and IECVELLSLASSSLN” (MSP4); “NDDSYRYDISEEIDD, DDSYRYDISEEIDDK, and DSYRYDISEEIDDKS” (Rh5); “CQGMYISLRSVHVHT, GMYISLRSVHVHTHN, and QGMYISLRSVHVHTH” (Ripr); “LVRGNYIGNINNIAR and RGNYIGNINNIARND” (Ron2); “MKSYISLFFILCVIF and SYISLFFILCVIFNK” (Sera5); and “VTLYQLKRVHSNMLI and LVTLYQLKRVHSNML” (Sera8). Nevertheless, only 10 epitopes were nominated to possess adequate antigenicity and no allergenicity with the capacity to induce IFN-*γ* cytokine, as shown in Table 6. In the following, population coverage analysis of all HTL epitopes revealed a high degree of allele coverage in West Africa (99.43%), Central Africa (99.21%), East Africa (99.17%), South America (98.51%), and South Asia (98.92%), with a global coverage rate of 97.17% (Table 7).

### 3.7. Immune Simulation

The major finding of the immune simulation was that Sera5 elicited the highest antibody titers, in particular IgM+IgG, IgM alone, and IgG1+IgG2, among other examined proteins. In the following, MSP1 and AMA-1 produced considerable specific antibodies. Such trend was observed regarding raised cytokines, with the predominance of IFN-*γ*, and memory T helper cells (Supplementary File 1).

## 4. Discussion

Generally said, routine vaccination programs annually protect the living of millions of people, whether residents or travelers, against several infectious diseases. Since the establishment of the germ theory, vaccination approaches have developed, from live-attenuated (rabies), killed (cholera, plague), and toxoid (diphtheria and tetanus) vaccines, to cell culture-based (polio) and recombinant (hepatitis B) vaccines [50]. Nevertheless, such conventional vaccinology modalities entail some drawbacks, so that they are costly and time-consuming, requiring well-equipped laboratories and a considerable labor demand [51]. Outstanding advances in the genomics and proteomics during last decades have improved our knowledge on their interconnected network and caused the emergence of a computer-based interdisciplinary branch of science, Bioinformatics, to finely categorize such a vast amount of information, which could be a major driving force towards research and development on next-generation vaccine design [52]. Using this method, the whole genome/proteome data of a given infectious agent and/or particular protein sequences involved in eliciting the immune responses, so-called immunogenic epitope, can be easily accessed, explored, and screened only using web-based servers and specialized computer programs. The outputs of such a fast and relatively reliable procedure could be utilized to construct rational vaccine candidates against various life-threatening infectious agents and subsequently validated through experimental approaches [53].

Malaria is a noxious parasitic disease among children and adults in the tropics and subtropics, and *P. falciparum* is the most pernicious species causing severe morbidity and mortality every year [54]. There is a robust pipeline of malaria vaccine candidates, being tested in preclinical and clinical trials, and most of which are recombinant-based vaccines that employed a single antigen [55]. However, development of a successful human malaria vaccine is a disputable issue, possibly due to the following [56]: (i) considerably large genome (23 mega base) consisting of 14 chromosomes, which encodes about 5300 genes; (ii) complex malaria life cycle, involving mosquito and human hosts; and (iii) remarkable genetic diversity and expression patterns within genomic and proteomic elements. To overcome such obstacles, utilization of bioinformatics tools is beneficial to specifically identify those epitopic regions with potential immunogenic capacity (immunodominant), in a rapid and authentic manner. In malaria, the major clinical sequelae and subsequent immune responses are attributed to the blood-stage parasites infecting and disrupting erythrocytes. Therefore, antigens expressed at this phase are important regarding malaria vaccine studies. Since RBCs do not express MHC-I molecules on their surface, a cytotoxic response could not be triggered *via* antigen presentation to T CD_8_^+^ cells [1]. However, antigens are actively transported by the parasite through the parasitophorous vacuole to the erythrocyte surface, where recognition by specialized B-cells occurs, leading to the production of high-affinity, class-switched anti-Plasmodia antibodies as the fundamental immune response against blood-stage parasites [57].

In present study, 14 blood-stage vaccine candidate antigens of Pf were enrolled in order to assess their primary structural and biochemical functions and to discover finely screened epitopic regions with potential affinity to B-cells and human MHC-II. In this sense, AMA1 is an interspecies common vaccine candidate in both liver and blood phases, with conserved function and structure among orthologs. This protein is expressed on merozoite surface in the blood-stage and play a role in RBC invasion [58]. Another significant protein, CyRPA, is a part of a conserved erythrocyte invasion complex along with Rh5 and Ripr and is a potential target for cross-strain neutralizing antibodies [59]. Another erythrocyte-binding protein is Eba175, which matches to its receptor, glycophorin A, as the major glycoprotein on human RBCs [60]. *In vitro* studies have shown that upon Eba175 inhibition, invasion of merozoites has been blocked [61]. Proteins of the MSP superfamily are another important antigens on merozoites; among these, MSP1 is a glycosylphosphatidylinositol-anchored protein and the most abundant member, whereas MSP3 and MSP6 are a distinct group without glycosylphosphatidylinositol anchor or transmembrane domain [62]. The ralp1 protein is especially expressed in schizonts stages, being localized to rhoptries and first evidenced as a vaccine candidate in 2013 [63]. RESA protein, previously known as Pf155, is secreted from dense granules to the parasitophorous vacuole and thereafter transferred to the inner RBC membrane. Reactive antibodies to RESA inhibit merozoite invasion [64] and RESA-based recombinant vaccines were shown to be protective in monkeys [65]. Currently, the most advanced blood-stage candidate is the rhoptry-based Rh5 which binds to basigin and this complex is vital for RBC invasion in all tested strains of Pf [66]. The Ripr is localized to the micronemes being composed of 10 epidermal growth factor-like domains. It is highly conserved, capable of inducing cross-strain neutralizing antibodies [67]. Moreover, Ron2, a bridging protein between AMA1 and moving junction [68], and Sera proteins have been recognized as potential vaccine candidates [69]. Altogether, targeted recognition of aforementioned antigens by antibodies should relatively or completely block merozoite invasion and lead to rapid phagocytosis of the parasites [70].

In the first step of this study, the amino acid sequences of 14 blood-stage Pf vaccine candidate antigens (AMA1, CyRPA, Eba175, MSP1, MSP3, MSP4, MSP6, Ralp1, RESA, Rh5, Ripr, Ron2, Sera5, and Sera8) were retrieved through UniProt KB and their antigenicity was assessed using VaxiJen v2.0 server. Pertinent to a 0.45 threshold score, all blood-stage proteins were shown to possess adequate antigenicity, and the highest scores belonged to two MSP proteins, i.e., MSP4 (1.0076) and MSP3 (0.8309). MSP3 is a soluble protein that forms a protein complex with MSP1, MSP6, and MSP7. Since 1994, it was discovered that MSP3-protective antibodies could be discovered in animals and humans. Later in 2003 and 2009, promising findings were obtained in preclinical and phase I clinical trial of the GMZ2 recombinant vaccine (containing MSP3 and glutamate-rich protein, GLURP), and further studies more emphasized on the potency of this MSP3-based vaccine candidates [71]. Previously, MSP4 was found to be immunogenic in nature both during natural infection and in laboratory animals, with limited gene polymorphism which simplifies the vaccine formulations [72]. A good vaccine candidate should not elicit allergenic reactions; hence, we performed allergenicity prediction for all 14 proteins using two web servers. Our results showed that half of the proteins, such as Eba175, MSP1, MSP4, MSP6, Ralp1, Rh5, and Ron2, were nonallergens based on AllergenFP and AllerTOP servers. All of the proteins had relatively high thermostability and hydrophilic in nature, based on aliphatic scores and negative GRAVY. Other significant biophysical features required for designing peptide-based vaccines are protein stability and solubility. Accordingly, Protein-Sol server demonstrated that all proteins are soluble, except of AMA1, Ripr, Ron2, and Sera8, and all molecules were unstable with the exception of AMA1, CyRPA, MSP1, Rh5, Sera5, and Sera8 [73].

In the second step, the presence of a signal peptide was predicted using SignalP online tool in all proteins but not in MSP4, MSP6, Ralp1, RESA, Rh5, and Ron2. Signal peptides destine a protein towards a secretory route and for different functions including secretory-excretory antigen, structural molecule, and/or as a virulence factor [74–76]. Based on deep TMHMM tool, only AMA1 and RESA had a single transmembrane domain. In the following, PTM prediction was accomplished for all proteins, displaying a varied number of PTM sites, including phosphorylation mostly in MSP1, Eba175, and Ron2 as well as N- and O-glycosylation as prominent PTMs in Eba175. Of note, palmitoylation was present in all blood-stage vaccine candidates, except of MSP3, MSP6, and Ron2. Recognition of PTM sites is of utmost importance when recombinant production of these proteins is of interest, since appropriate eukaryotic machineries (e.g., yeast, insect, or mammalian) should be preferred as a replacement for bacterial expression systems [77].

Structural analysis of the proteins is a critical step regarding multiepitope vaccine design. In this study, the secondary structure was determined for each submitted protein sequence, rendering random coils as the most frequent secondary structure, followed by alpha helix. Random coils are mostly located at the surface of a protein as protruding structures and such flexible regions may provide possible evidence of epitope identification [78]. Inner structures like alpha helices, also, maintain the whole conformation of a protein while interacting with other molecules [79]. Furthermore, the 3D structure of those stable proteins were predicted using SWISS-MODEL homology modeling server.

Humoral immune responses are the principal tool to combat the blood-stage merozoites of malaria [57]. Although it is said that produced antibody titers are only transiently present in the circulation of affected individuals and decline by next transmission season [80], serological evidences imply that there exists inverse correlation between elevated breadth of antibody specificity and a lower chance of experiencing clinical episodes and a subsequent hospital admission in severe Pf infection [81]. Protective malaria-specific antibodies may ignite effector functions such as merozoite opsonization, phagocytosis, inhibition of cell invasion, respiratory burst activation, and complement-dependent parasite killing [82]. Due to the importance of antibody responses in malaria infection, as the final and effector products of B-cell responses, we predicted potential B-cell epitopes in 14 blood-stage Pf vaccine candidates and screened those shared epitopes in terms of antigenicity, allergenicity, and water solubility. Based on our results, potential B-cell epitopes that were highly antigenic, water soluble, and without allergenicity were predicted in all proteins, most frequently in MSP1, Eba175, Ralp1, Ron2, and Sera proteins, which could be further utilized to construct multiepitope vaccine candidates. The predicted linear B-cell epitopes for AMA1 embedded in its pro-domain_24-97_ (HQEHTYQQED_56-65_), DI domain_98-305_ (NMIPDNDKNSN_223-233_), and DII domain_305-442_ (LPTGAFKADRYKS_380-392_). Our finding is in line with previous studies that remark major targets for malaria inhibitory responses present in DI and DII domains of AMA1 [83]. Also, the serine-rich region of Sera5 antigen is intrinsically unstructured, and it was shown to possess strong antigenicity with protective epitopes [84], as evidenced here in our study (SSSSSSSSSSSSSE_221-234_). Altogether, Pf blood-stage development can be inhibited in multiple ways by specific antibodies, including direct inhibition of merozoite invasion and/or intraerythrocytic development or through recalling immune effector cells [85].

In the following, CD_4_^+^ T-cell responses have been shown to be associated with control of blood-stage parasites; also, IFN-*γ* has been demonstrated as a potent modulator in harnessing acute infections in rodent models [86]. In the current in silico study, 24 out of 70 predicted epitopes were capable to induce IFN-*γ*; however, only 10 epitopes were actually antigenic in nature, without allergenicity, and were potent IFN-*γ* inducers, mostly in Sera5, Ripr, and Eba175 proteins. Additionally, we performed population coverage analysis for the predicted 70 HTL epitopes for blood-stage Pf proteins and showed that a high degree of allele coverage presents in West Africa (99.43%), Central Africa (99.21%), East Africa (99.17%), South America (98.51%), and South Asia (98.92%), with a global coverage rate of 97.17%. Such a good rate of allele coverage is beneficial in future vaccine design strategies in such populations. Notably, Sera5 showed considerable antibody, cytokine, and memory T helper cell production, as shown by in silico simulation; high-level antibody titers against Sera5 have been detected in those individuals inhabiting malaria-endemic areas [87]. This protein is the most robustly expressed antigen among family members in both trophozoite and schizonts stages [88]. Agglutination of merozoites and ruptured schizonts have shown to be enhanced by such antibodies *in vitro* [87]. Immunization using AMA1 has shown good protection against the infection in *in vitro* growth inhibition assays and experimental animal models through production of specific antibodies [83]. Moreover, MSP1 could significantly elicit the immune responses, as evidenced in previous studies [89–91].

## 5. Conclusion

In a nutshell, malaria is a devastating parasitic infection with global prevalence, mostly in tropics and subtropics. Despite huge efforts in the field of vaccination, a successful human malaria vaccine is yet to be elucidated. Computer-aided advances have led to the credible and reproducible in silico methods for rational vaccine design in a time- and cost-effective manner. We predicted some preliminary biochemical features along with carefully screened HTL and B-cell epitopes of 14 blood-stage vaccine candidate antigens of Pf. Such information is critically required for subunit (DNA/protein vaccines) and multiepitope vaccine design, which can be further formulated and evaluated using different delivery platforms. It is, also, noteworthy that three steps must be considered in such processes, including (1) choosing appropriate modality for protein/peptide analysis, (2) multiserver prediction using different machine learning techniques for a more robust analysis, and (3) wet lab experiments to validate the output of *in silico* analysis. Here, we demonstrated different bioinformatics features of blood-stage antigens, mostly in those stable proteins (AMA1, CyRPA, Rh5, MSP1, and Sera5), and showed that among stable proteins Sera5 possessed the dramatically highest antibody titer production, cytokine increase (in particular IFN-*γ*), and helper memory cell elevation among others. Since AMA1 is a key molecule for merozoite invasion of erythrocytes [92] and sporozoite penetration in hepatocytes [93], targeting this protein, mostly those epitopes in the DI and DII domains, may be beneficial in prevention of preerythrocytic and intraerythrocytic stages. Also, PfMSP1 is a highly antigenic vaccine candidate with promising results based on partial or complete protein sequences. According to the findings represented here, future studies on the multiepitope vaccine design should particularly emphasize on the Sera5, along with MSP1 and AMA-1 vaccine candidates, which have shown potential antigenicity and immunogenicity.

## Figures and Tables

**Table 1:** Prediction of antigenicity, allergenicity, solubility, stability, and physicochemical properties of 14 *P. falciparum* erythrocytic stage proteins.

Server/Parameter	AMA1	CyRPA	Eba175	MSP1	MSP3	MSP4	MSP6	Ralp1	RESA	Rh5	Ripr	Ron2	Sera5	Sera8
VaxiJen score	0.5639	0.5732	0.6903	0.6039	0.8309	1.0076	0.6790	0.6627	0.5626	0.4882	0.4980	0.5071	0.7150	0.6148
AllergenFP	No	No	No	No	No	No	No	No	Yes	No	No	No	Yes	Yes
AllerTOP	Yes	Yes	No	No	Yes	No	No	No	Yes	No	Yes	No	No	No
Protein-Sol	0.415	0.545	0.505	0.523	0.806	0.894	0.778	0.613	0.714	0.503	0.445	0.400	0.501	0.302
No. of AA	622	362	1435	1639	394	272	427	677	1073	526	1086	1369	997	690
MW	72009.78	42775.68	167390.05	187618.81	45134.89	30548.52	48448.28	79640.22	104906.84	62995.86	125892.52	158975.66	111768.15	80850.47
pI	5.55	5.39	5.62	5.98	4.55	4.57	4.57	5.9	4.46	8.62	6.36	9.37	5.26	8.46
+ residues (Arg+Lys)	76	43	221	234	58	35	47	105	109	83	127	202	109	96
- residues (Asp+Glu)	96	54	258	251	104	65	91	115	243	76	136	151	141	86
Half-life (mammalian reticulocytes)	30 hours	30 hours	30 hours	30 hours	30 hours	30 hours	30 hours	1.1 hours	30 hours	30 hours	30 hours	30 hours	30 hours	30 hours
Instability index	37.10 (stable)	39.20 (stable)	42.42 (unstable)	39.18 (stable)	52.98 (unstable)	43.52 (unstable)	54.73 (unstable)	48.53 (unstable)	51.86 (unstable)	35.04 (stable)	42.59 (unstable)	45.52 (unstable)	37.89 (stable)	36.08 (stable)
Aliphatic index	62.88	88.23	62.67	85.55	57.82	67.65	63.47	64.02	74.04	83.38	70.87	79.39	65.75	69.04
GRAVY	-0.802	-0.330	-1.072	-0.691	-1.354	-0.969	-1.257	-1.302	-0.930	-0.831	-0.607	-0.523	-0.706	-0.757
SignalP	0.9996 (yes)	0.9998 (yes)	0.637 (yes)	0.9987 (yes)	0.693 (yes)	0.266 (no)	0.3255 (no)	0.0062 (no)	0.0001 (no)	0.1967 (no)	0.9997 (yes)	0 (no)	0.9996 (yes)	0.9996 (yes)
TMHMM	1	—	—	—	—	—	—	—	1	—	—	—	—	—
Subcellular localization	CM: 0.7860 ER: 0.6861	EX: 0.8793	CM: 0.5714	CM: 0.7836	EX: 0.8742 CM: 0.5912	CM: 0.8955 EX: 0.6376	CM: 0.7187	CM: 0.4710	ER: 0.3461	CM: 0.6154	EX: 0.6184	Mit: 0.5620	EX: 0.7846 CM: 0.6174	EX: 0.8235

**Table 2:** Prediction of the posttranslational modification sites within 14 *P. falciparum* erythrocytic stage protein sequences.

PTM	AMA1	CyRPA	Eba175	MSP1	MSP3	MSP4	MSP6	Ralp1	RESA	Rh5	Ripr	Ron2	Sera5	Sera8
Phosphorylation														
*S*	28	13	89	103	22	18	27	25	51	19	45	90	87	37
**T**	12	8	52	45	13	4	16	18	33	19	23	33	39	19
Y	25	10	28	39	9	3	3	14	22	15	38	33	17	25
Palmitoylation	2	1	2	3	—	1	—	2	2	1	4	—	3	5
Lysine acetylation	4	1	27	31	64	35	37	36	33	11	5	44	10	4
N-Glycosylation	4	3	15	11	4	2	2	4	4	2	11	13	2	2
O-Glycosylation	3	—	120	72	25	22	31	16	15	5	21	12	58	8

*S*: serine phosphorylation; **T**: tyrosine phosphorylation; Y: threonine phosphorylation.

**Table 3:** Prediction results for secondary structures of 14 selected erythrocytic stage *P. falciparum* antigens, using Garnier–Osguthorpe–Robson (GOR IV) server.

Secondary structure	AMA1	CyRPA	Eba175	MSP1	MSP3	MSP4	MSP6	Ralp1	RESA	Rh5	Ripr	Ron2	Sera5	Sera8
Alpha helix	139 (22.35)	29 (8.01)	428 (29.83)	789 (48.14)	278 (70.56)	100 (36.76)	138 (32.32)	275 (40.62)	544 (50.70)	210 (39.92)	106 (9.76)	667 (48.72)	201 (20.16)	136 (19.71)
Extended strand	91 (14.63)	134 (37.02)	236 (16.45)	179 (10.92)	34 (8.63)	46 (16.91)	55 (12.88)	95 (14.03)	109 (10.16)	74 (14.07)	318 (29.28)	175 (12.78)	224 (22.47)	171 (24.78)
Random coil	392 (63.02)	199 (54.97)	771 (53.73)	671 (40.94)	82 (20.81)	126 (46.32)	234 (54.8)	307 (45.35)	420 (39.14)	242 (46.01)	662 (60.96)	527 (38.5)	572 (57.37)	383 (55.51)

**Table 4:** Prediction of shared continuous B-cell epitopes in 14 selected erythrocytic stage *P. falciparum* antigens with subsequent screening regarding antigenicity, allergenicity, and water solubility.

Protein	Shared epitopes	VaxiJen antigenicity	AllergenFP allergenicity	PepCalc water solubility
AMA1	DHEGAEPAPQEQNL	0.3882	No	Good
	KMDEPQHYG	1.0432	Yes	Good
	EEYKDEYADI	0.6827	Yes	Good
	LYIAAQENNGPRY	0.3357	No	Poor
	**HQEHTYQQED**	0.6384	No	Good
	**LPTGAFKADRYKS**	1.3353	No	Good
	FGRGQNYWEHPYQ	-0.2076	No	Poor
	**NMIPDNDKNSN**	1.1296	No	Good
	TEYMAKYDIE	0.2234	No	Good
CyRPA	**SPYKFKDDNKKD**	1.0096	No	Good
	DNINNVKNV	0.0707	Yes	Good
	**NDLFKETDLTG**	1.2358	No	Good
	**EEFSNRKKDMT**	0.8601	No	Good
	KDNKVLQDVSTL	0.1229	No	Good
	LKDKLLSSYVSLPL	0.9588	No	Poor
Eba175	**HHGNRQDRGGNSGN**	2.2151	No	Good
	**REDERTLTKEYEDI**	0.7318	No	Good
	**GPKGNEQKERDDDS**	1.5703	No	Good
	**SRINNGRNTSS**	1.5214	No	Good
	**SINNGDDSGSGS**	1.3930	No	Good
	SNNNNFNNIPS	-0.4858	No	Poor
	EKFNELDKKKYGN	-0.0866	No	Good
	**YCDCKHTTTLVK**	0.9283	No	Good
	**EEYNKQAKQYQEYQ**	0.8479	No	Good
	NTLHLKDIRNEE	0.4600	No	Good
	GDDSGSGSAT	2.1372	Yes	Good
	**NPEDRNSNTLHLK**	0.9643	No	Good
	NVQQSGGIVN	1.1086	No	Poor
	**KAEEERLSHTDI**	0.5411	No	Good
MSP1	**VPTPPAPVNNKTEN**	0.6756	No	Good
	NPPPANSGNTPNTL	0.1965	No	Poor
	**PPVPVPVPEAK**	0.6912	No	Good
	GCFRHLDEREE	-0.0243	No	Good
	EQLFEKKIT	0.5568	Yes	Good
	**NPSDNSSDSDAK**	1.6344	No	Good
	**LEQSQPKKPASTHV**	0.7679	No	Good
	**ILKQYKITKEEESK**	0.8440	No	Good
	**DKNKKIEEHEKE**	1.3583	No	Good
	LNSLNNPKH	0.2690	Yes	Good
	SSNYVVKDPYKFLN	0.2960	No	Good
	**VPNSYKQENK**	0.6840	No	Good
	**LKHNIHVPNSYKQE**	0.6190	No	Good
MSP3	YKKAKEASQDAEKA	0.9912	Yes	Good
	ENVNTPIVGNSM	0.9144	Yes	Poor
	LGWEFGGGVPEHKK	0.4004	Yes	Good
	**NNNSQIENEEK**	1.3218	No	Good
MSP4	**ASEQGEESHKKE**	1.6269	No	Good
	HVGEEEDHNEGEGE	1.6106	Yes	Good
	VVHFFIICTINF	-0.0159	Yes	Poor
	KKKTEAIPKKVVQ	0.0166	No	Good
	SDAAEKKDEKEASE	1.1597	Yes	Good
	**DDDEDDDTYN**	1.4465	No	Good
MSP6	SDIQATYQFPPTP	-0.4445	Yes	Poor
	GTRGIHTYSGES	0.1113	No	Good
	**AEENSETNKNPT**	1.1044	No	Good
	PPTPGRIINPR	0.8340	Yes	Good
	**EEEKKEEEEKKE**	1.8461	No	Good
	DKKTEYLLEQI	0.4517	Yes	Good
Ralp1	IVYNNIQGQG	0.9383	Yes	Poor
	NNIQGQGELLQ	1.0569	Yes	Poor
	**GNGDNESSQRVD**	1.5302	No	Good
	NVEKKSNMESVNNN	0.5877	Yes	Good
	NANKVPNVAH	-0.2316	Yes	Poor
	**QSDDITDEQKKY**	0.8375	No	Good
	**KLDQQGELKNVSVV**	0.8150	No	Good
	**KDNTFNFHKN**	0.5660	No	Good
	NKDNTFNFHKN	0.2417	No	Good
	**DENNNTNDKNNC**	1.3952	No	Good
RESA	AEMKKRAQK	1.4690	Yes	Good
	**PQQEEPVQTVQE**	0.7672	No	Good
	PYADSENPIVV	0.6307	Yes	Good
	**NVEENVEENVEENV**	1.3997	No	Good
	**NINSNVDNGN**	1.6573	No	Good
	TQANKQELANI	0.1906	No	Good
	YGYDGIKQV	-1.0965	Yes	Good
	RWYNKYGYDGIKQV	-0.4537	No	Good
	SSSSGVQFTDRCS	0.1913	Yes	Good
	KDFTGTPQIVTLLR	-0.0496	No	Good
	NLYGETLPVNPY	1.8128	Yes	Poor
Rh5	AIKKTKNQEN	0.6552	Yes	Good
	**TEEEKDDIKNGKDI**	1.2106	No	Good
	SCYNNNFCNTNG	-0.0212	No	Poor
	**NNKNDDSYRYD**	0.7851	No	Good
	**IINDKTKIIQD**	0.9855	No	Good
	SKNLNKDLSD	0.5424	Yes	Good
	KKLEHPYDINNK	-0.5249	No	Good
	SVFNQINDGMLLN	0.2222	No	Poor
	KKINETYDKV	-0.4121	Yes	Good
Ripr	**TDPHTNSNNI**	1.1210	No	Good
	KEKFYKNNLY	1.3379	Yes	Good
	CYKKTFCGVVIP	0.9010	No	Poor
	EIQNEISSHNSNQF	0.4284	No	Good
	SVLCSQNQVCQ	0.0578	No	Poor
	HLISRNSR	0.2658	Yes	Good
	CANDYKMEDGI	-0.0054	No	Good
Ron2	**YASKQTSDSDDSDI**	1.1198	No	Good
	**NDSSEESAKKKLQD**	0.6400	No	Good
	**WLEFNDNPTNASS**	0.5370	No	Good
	**KNDTSEKSSQKN**	1.1045	No	Good
	PKRTTTFYGERRL	0.1554	No	Good
	**DSKGMLARQIFTKG**	1.1186	No	Good
	**RPKRTTTFYGE**	1.4781	No	Good
	RNPGMMAI	0.1053	Yes	Poor
	FSTYMGFDRRSFLP	-0.3423	No	Poor
	REQIENFK	0.0655	Yes	Good
Sera5	**SSSSSSSSSSSSSE**	1.8405	No	Good
	GASPQGSTG	1.5737	No	Poor
	PEDKDNKGK	2.1879	Yes	Good
	**TVSVSQTSTSSEKQ**	0.7225	No	Good
	**PANGPDSPTVKPP**	0.6062	No	Good
	**TKDTTENNKVDV**	0.9430	No	Good
	**LPSNGTTGEQ**	1.2054	No	Good
	**PMNNKTTKKESKIY**	0.8272	No	Good
	KILHNKNEPNSL	0.1980	No	Good
	KTNNAISFESNSGS	0.3549	No	Good
	VNKRGLVLPELNY	0.2452	No	Good
	EDDDEDDYTEY	1.2934	Yes	Good
	**YNYVKVGEQCPKVE**	0.8915	No	Good
Sera8	**DAYYDNNDDY**	0.8582	No	Good
	**NMAHNPIDVPLPND**	0.6719	No	Good
	FSFEKDSDT	1.1152	Yes	Good
	DKLTFDHDGT	-0.0833	No	Good
	**NYKDNYENANNH**	0.6804	No	Good
	QYDKNSNDYDRN	0.8916	Yes	Good
	**SKRKDEYKEPYS**	0.8054	No	Good
	IYHGYFKVSFK	0.9309	Yes	Poor
	**PITGSECPDN**	1.2238	No	Good
	**INEENNMEHMKD**	0.7345	No	Good

**Table 5:** Prediction of conformational B-cell epitopes in five modeled erythrocytic stage *P. falciparum* antigens (AMA1, CyRPA, MSP1, Rh5, and Sera5).

Protein	No.	Residues	No. of Residues	Score
AMA1	1	A:H200, A:K203, A:D204	3	0.985
	2	A:G107, A:N108, A:P109, A:W110, A:T111, A:E112, A:Y113, A:M114, A:K305, A:N306, A:L307, A:Q308, A:N309, A:A310, A:K311, A:G313, A:L314, A:W315, A:V316, A:D317, A:G318, A:N319, A:C320, A:E321, A:D322, A:I323, A:P324, A:H325, A:E328, A:F329, A:S330, A:A331, A:I332, A:D333, A:Y402, A:N403, A:T404, A:E405, A:T406, A:Q407, A:K408, A:N413, A:V414, A:K415, A:P416, A:T417, A:C418, A:L419, A:I420, A:N421, A:N422, A:S423, A:S424, A:Y425, A:I426, A:E436, A:V437	57	0.731
	3	A:P184, A:P185, A:T186, A:E187, A:P188, A:L189, A:M190, A:S191, A:P192, A:M193, A:T194, A:L195, A:D196, A:E197, A:M198, A:R199, A:F201, A:Y202, A:N205, A:K206, A:Y207, A:V208, A:K209, A:N210, A:L211, A:D212, A:D242, A:K243, A:D244, A:K245, A:K246, A:N286	32	0.699
	4	A:V169, A:A170, A:T171, A:G172, A:N173, A:Q174	6	0.673
	5	A:E136, A:V137, A:A138, A:G139, A:T140, A:Q141, A:N160, A:S161, A:N162, A:T163, A:T164, A:L176, A:K177, A:G179, A:G222, A:N223, A:M224, A:I225, A:P226, A:D227, A:N228, A:D229, A:K230, A:N231, A:S232, A:N233, A:N257, A:N258, A:G259, A:P260, A:R261, A:Y262, A:C263, A:N264, A:K265, A:D266, A:E267, A:S268, A:K269, A:R270, A:N271, A:S272, A:M273, A:F274, A:E354, A:Q355, A:H356, A:L357, A:T358, A:D359, A:Y360, A:E361, A:K368, A:N369, A:K370, A:N371, A:A372, A:S373, A:M374, A:I375, A:K376, A:S377, A:A378, A:F379, A:L380, A:P381, A:T382, A:G383, A:A384, A:F385, A:K386, A:A387, A:D388, A:R389, A:K391	75	0.672
	6	A:V326, A:N327, A:E410	3	0.636
CyRPA	1	A:K186, A:K188, A:D189, A:D190, A:N191, A:K192, A:K193, A:D194, A:D195, A:V213, A:K215, A:D217, A:N218, A:Y219, A:K220, A:L221, A:G222, A:V223, A:Q224, A:Y225, A:G244, A:D245, A:N246, A:I247, A:N248, A:N249, A:V250, A:T259, A:H260, A:E261, A:K262, A:D263, A:L264, A:E265, A:V267	35	0.743
	2	A:P184, A:Y185, A:F187	3	0.732
	3	A:R31, A:H32, A:V33, A:F34, A:R36, A:T37, A:E38, A:R271, A:D272, A:F273, A:L274, A:K275, A:D276, A:N277, A:K278, A:N287, A:D288, A:E289, A:N296, A:D297, A:N298, A:N299, A:F300, A:A301, A:E302, A:Y304, A:N308, A:N309, A:E310, A:N311, A:S312, A:I313, A:L314, A:K316, A:P317, A:E318, A:Y320, A:G321, A:N322, A:T323, A:T324, A:A325, A:G326, A:I335, A:D336, A:E337, A:N338, A:R339, A:T355, A:I356, A:Y357, A:Y358, A:A359, A:N360, A:Y361	55	0.696
	4	A:K170, A:I171, A:E172, A:N173, A:N235	5	0.694
	5	A:D67, A:L68, A:K69, A:G70, A:E71, A:E72, A:D73, A:E74, A:T75, A:H76, A:I88, A:T89, A:L90, A:N91, A:D92, A:L93, A:F94, A:K95, A:E96, A:T97, A:D98, A:L99, A:T100, A:G101, A:R102, A:D110, A:V111, A:E112, A:E113, A:E120, A:D121, A:E122, A:E123, A:F124, A:S125, A:N126, A:K128, A:K129, A:D130, A:M131, A:T132, A:Y137, A:S138, A:N139, A:D140, A:G141, A:K142, A:E143, A:Y144, A:N145, A:N146, A:S147, A:E148, A:I149, A:T150, A:I151, A:S152, A:D153	58	0.671
	6	A:K43, A:N44, A:N45, A:V46, A:P47, A:C48, A:V83, A:K84, A:D85, A:S86, A:S347, A:Q348, A:G349, A:I350, A:Y351	15	0.589
	7	A:R174, A:H202, A:D203, A:K204, A:G205, A:E206, A:T207, A:W208	8	0.582
MSP1	1	A:Q720, A:A721, A:G722, A:S723, A:A724, A:L725	6	0.806
	2	A:I430, A:N431, A:P432, A:F433, A:D434, A:Y435, A:E438, A:P439, A:S440, A:K441, A:N442, A:I443, A:Y444, A:T445, A:D446, A:N447, A:E448, A:R449, A:K450, A:K451, A:F452, A:I453, A:N454, A:E455, A:I456, A:K457, A:E458, A:K459, A:L547, A:K548, A:M550, A:E551, A:D552, A:Y553, A:S554, A:L555, A:R556, A:N557, A:I558, A:V559, A:V560, A:E561, A:K562, A:E563, A:L564, A:K565, A:Y566, A:Y567, A:K568, A:N569, A:L570, A:I571, A:S572, A:K573, A:I574, A:E575, A:N576, A:E577, A:I578, A:E579, A:T580, A:L581, A:V582, A:E583, A:N584, A:I585, A:K586, A:K587, A:D588, A:Q591, A:L592, A:E594, A:K595, A:K596, A:I597, A:T598, A:K599, A:D600, A:E601, A:N602, A:K603, A:P604, A:D605, A:E606, A:K607, A:I608, A:L609, A:E610, A:V611, A:S612, A:D613, A:I614, A:V615, A:K616, A:V617, A:Q618, A:V619, A:Q620, A:K621, A:V622, A:L623, A:L624, A:M625, A:N626, A:K627, A:I628, A:D629, A:E630, A:L631, A:K632, A:T634, A:Q635, A:I637, A:L638, A:V641, A:E642, A:K644, A:H645, A:N646, A:M852, A:E853, A:I854, A:Y855, A:E856, A:K857, A:E858, A:M859, A:V860	128	0.759
	3	A:K689, A:K690, A:N691, A:I692, A:K693, A:T694, A:E695, A:G696, A:Q697, A:S698, A:D699, A:N700, A:S701, A:E702, A:P703, A:S704, A:T705, A:E706, A:G707, A:E708, A:I709, A:T710, A:G711, A:Q712, A:A713, A:T714, A:T715, A:K716, A:P717, A:G718, A:Q719, A:E726, A:G727, A:D728, A:S729, A:V730, A:Q731, A:A732, A:Q733, A:A734, A:Q735, A:E736, A:Q737, A:K738, A:Q739, A:A740, A:Q741, A:P742, A:P743, A:V744, A:P745, A:V746, A:P747, A:V748, A:P749, A:E750, A:A751, A:K752, A:A753, A:Q754, A:V755, A:P756, A:T757, A:P758, A:P759, A:A760, A:P761, A:V762, A:N763, A:N764, A:K765, A:T766, A:E767, A:N768, A:V769	75	0.756
	4	A:M51, A:V52, A:L53, A:N54, A:E55, A:G56, A:T57, A:S58, A:G59, A:T60, A:A61, A:V62, A:T63, A:T64, A:S65, A:T66, A:P67, A:G68, A:S69, A:K70, A:G71, A:S72, A:V73, A:A74, A:S75, A:G76, A:G77, A:S78, A:G79, A:G80, A:S81, A:V82, A:A83, A:S84, A:G85, A:G86, A:S87, A:V88, A:A89, A:S90, A:G91, A:G92, A:S93, A:V94, A:A95, A:S96, A:G97, A:G98, A:S99, A:V100, A:A101, A:S102, A:G103, A:G104, A:S105, A:G106, A:N107, A:S108, A:R109, A:R110, A:T111, A:N112, A:P113, A:S114, A:D115, A:N116, A:S117, A:S118, A:D119, A:S120, A:D121, A:A122, A:K123, A:S124, A:Y125, A:T156, A:L157, A:C158, A:D159, A:N160, A:I161, A:H162, A:G163, A:K165, A:Y166, A:D233, A:N234, A:V235, A:G236, A:K237, A:E239, A:D240, A:I242, A:K243, A:K244, A:N245, A:K246, A:K247, A:T248, A:I249, A:E250, A:N251, A:I252, A:N253, A:E254, A:L255, A:I256, A:E257, A:E258, A:S259, A:K260, A:K261, A:T262, A:I263, A:D264, A:K265, A:N266, A:K267, A:N268, A:A269, A:T270, A:K271, A:E272, A:E273, A:E274, A:K275, A:K276, A:K277, A:L278, A:Y279, A:Q280, A:A281, A:Q282, A:D284, A:L285, A:Y288, A:I306, A:L309, A:K311, A:N312, A:E313, A:N314, A:I315, A:K316, A:E317, A:L318, A:L319, A:D320, A:K321, A:I322, A:N323, A:E324, A:I325, A:K326, A:N327, A:P328, A:P329, A:P330, A:A331, A:N332, A:S333, A:G334, A:N335, A:T336, A:P337, A:N338, A:T339, A:L340, A:L341, A:D342, A:K343, A:N344, A:K345, A:K346, A:I347, A:E348, A:E349, A:H350, A:E351, A:K352, A:E353, A:I354, A:E356, A:I357, A:D505	185	0.737
	5	A:I460, A:K461, A:I462, A:E463, A:K464, A:K465, A:K466, A:I467, A:E468, A:S469, A:D470, A:K471, A:K472, A:S473, A:Y474, A:E475, A:D476, A:R477, A:S478, A:K479, A:S480, A:N482, A:D483	23	0.62
Rh5	1	A:K247, A:D249, A:S251, A:Y252, A:Y254, A:D255, A:I256, A:S257, A:E258, A:E259, A:I260, A:D261, A:D262, A:K263, A:S264, A:E265, A:E266, A:T267, A:D268, A:D269, A:E270, A:T271, A:E272, A:E273, A:V274, A:E275, A:D276, A:S277, A:I278, A:Q279, A:D280, A:T281, A:D282, A:S283, A:N284, A:H285, A:T286, A:P287, A:S288, A:N289, A:K290, A:K291, A:K292, A:N293, A:D294, A:L295, A:M296, A:N297	48	0.822
	2	A:Y242, A:N375, A:N377, A:K378, A:L380, A:S381, A:D382, A:T384, A:N385, A:I386, A:L387, A:Q388, A:Q389, A:S390, A:E391, A:L392, A:L393, A:L394, A:T395, A:N396, A:L397, A:N398, A:K399, A:K400, A:M401, A:F494, A:H495, A:H496, A:L497, A:I498, A:Y499, A:V500, A:L501, A:Q502, A:M503, A:K504	36	0.757
	3	A:S192, A:I193, A:Y194, A:H195, A:K196, A:S197, A:S198, A:T199, A:Y200, A:G201, A:K202, A:C203, A:I204, A:A205, A:V206, A:D207, A:A208, A:F209, A:K211, A:K212, A:E215, A:K327, A:M330, A:D331, A:K333, A:N334, A:Y335, A:T337, A:N338, A:L339, A:F340, A:E341, A:Q342, A:L343, A:S344, A:C345, A:Y346, A:N347, A:N348, A:N349, A:F350, A:C351, A:N352, A:T353, A:N354, A:R357, A:Y358, A:E362, A:Y363, A:K436, A:I437, A:Q439, A:D440, A:K441, A:I442, A:K443, A:L444, A:N445, A:I446, A:W447, A:R448, A:T449, A:F450, A:Q451, A:K452, A:D453, A:E454, A:L455, A:L456, A:K457, A:R458	71	0.688
	4	A:K316, A:K319, A:N320, A:H321, A:E322, A:N323, A:D324, A:N326	8	0.62
	5	A:D361, A:H365, A:L369, A:K372	4	0.592
	6	A:G402, A:S403, A:Y404	3	0.55
Sera5	1	A:D393, A:D394, A:D395, A:E396, A:D397, A:D398, A:Y399, A:T400, A:E401	9	0.784
	2	A:Y402, A:K403, A:T405, A:E406, A:S407, A:I408, A:D409, A:N410, A:I411, A:L412, A:V413, A:K414, A:M415, A:F416, A:K417, A:T418, A:N419, A:E420, A:N421, A:N422, A:D423, A:K424, A:S425, A:E426, A:L427, A:I428, A:K429, A:L430, A:E431, A:E432, A:V433, A:D434, A:D435, A:S436, A:L437, A:K438, A:L439, A:E440, A:L441, A:M442, A:N443, A:C445, A:S446, A:L447, A:K449, A:D450, A:G461, A:M462, A:G463, A:N464, A:E465, A:M466, A:D467, A:I468, A:F469, A:N470, A:K473, A:F491, A:L498, A:K499	60	0.778
	3	A:E515, A:L516, A:N517, A:Y518, A:D519, A:L520, A:E521, A:Y522, A:F523, A:N524, A:E525, A:H526, A:L527, A:Y528, A:N529, A:D530, A:K531, A:N532, A:S533, A:P534, A:E535, A:D536, A:K537, A:D538, A:N539, A:K540, A:G541, A:K542, A:G543, A:V544, A:V545, A:H546, A:V547, A:D548, A:T549, A:T550, A:L551, A:D555, A:T556, A:L557, A:S558, A:Y559, A:D560, A:N561, A:S562, A:D563, A:N564, A:M565, A:F566, A:C567, A:N568, A:K569, A:E570, A:D577, A:E578, A:N579, A:N580, A:M611, A:K612, A:G613, A:Y614, A:E615, A:P616, A:T617, A:S620, A:Y623, A:N626, A:C627, A:Y628, A:K629, A:G630, A:E631, A:H632, A:K633, A:D634, A:R635, A:C636, A:D637, A:E638, A:Q647, A:E650, A:D651, A:Y652, A:G653, A:F654, A:L655, A:P656, A:A657, A:E658, A:S659, A:N660, A:Y661, A:P662, A:N664, A:V666, A:K667, A:V668, A:G669, A:E670, A:Q671, A:C672, A:P673, A:K674, A:V675, A:E676, A:D677, A:H678, A:W679, A:M680, A:N681, A:L682, A:W683, A:D684, A:N685, A:G686, A:K687, A:I688, A:L689, A:H690, A:N691, A:K692, A:N693, A:E694, A:P695, A:N696, A:S697, A:K701, A:R710, A:N714, A:D716, A:K720, A:N771, A:Y772, A:V773, A:N774, A:S775, A:E776, A:G777, A:E778, A:K779, A:K780, A:N821, A:V822, A:D823, A:L824, A:P825, A:M826, A:N827	148	0.692
	4	A:N753, A:C755, A:G805, A:P806, A:T807, A:H808, A:C809, A:H810, A:F811	9	0.564
	5	A:S480, A:E481, A:E482, A:N483, A:I484	5	0.553

**Table 6:** Helper T lymphocyte-specific epitope prediction in 14 selected erythrocytic stage *P. falciparum* antigens and subsequent screening regarding IFN-*γ* induction, antigenicity, and allergenicity.

Protein	Allele	HTL epitope	Method	Percentile rank	VaxiJen antigenicity	AllerTOP allergenicity	IFN-*γ* inducer	
							Result	Score
AMA1	HLA-DPA1^∗^02 : 01/DPB1^∗^14 : 01	KMKIIIASSAAVAVL	NetMHCIIpan	0.03	0.7487	No	Negative	5
	HLA-DPA1^∗^02 : 01/DPB1^∗^14 : 01	DKMKIIIASSAAVAV	NetMHCIIpan	0.06	0.6712	No	Negative	5
	HLA-DRB1^∗^13 : 02	MKIIIASSAAVAVLA	NetMHCIIpan	0.07	0.7198	No	Negative	7
	HLA-DRB1^∗^09 : 01	YDKMKIIIASSAAVA	NetMHCIIpan	0.08	0.6072	Yes	Negative	5
	HLA-DQA1^∗^05 : 01/DQB1^∗^03 : 01	MKIIIASSAAVAVLA	Consensus (comb.lib./smm/nn)	0.09	0.7198	No	Negative	7
CyRPA	HLA-DRB3^∗^02 : 02	ELSFIKNNVPCIRDM	NetMHCIIpan	0.01	0.0131	Yes	Negative	-0.2933
	HLA-DQA1^∗^05 : 01/DQB1^∗^02 : 01	GRPHIFAYVDVEEII	Consensus (comb.lib./smm/nn)	0.01	-0.2266	No	Positive	0.8518
	HLA-DQA1^∗^05 : 01/DQB1^∗^02 : 01	HIFAYVDVEEIIILL	Consensus (comb.lib./smm/nn)	0.01	0.2274	Yes	Negative	12
	HLA-DRB3^∗^02 : 02	LSFIKNNVPCIRDMF	NetMHCIIpan	0.01	0.1361	Yes	Negative	-0.7208
	HLA-DQA1^∗^05 : 01/DQB1^∗^02 : 01	PHIFAYVDVEEIIIL	Consensus (comb.lib./smm/nn)	0.01	0.1163	No	Negative	3
Eba175	HLA-DPA1^∗^01 : 03/DPB1^∗^02 : 01	CNISIYFFASFFVLY	Consensus (comb.lib./smm/nn)	0.01	0.9694	Yes	Positive	1
	HLA-DRB1^∗^12 : 01	IFKFLITNKIYYYFY	Consensus (smm/nn)	0.01	0.1789	Yes	Negative	1
	HLA-DPA1^∗^01 : 03/DPB1^∗^02 : 01	**ISIYFFASFFVLYFA**	Consensus (comb.lib./smm/nn)	0.01	1.5296	No	Positive	1
	HLA-DPA1^∗^01 : 03/DPB1^∗^02 : 01	**KCNISIYFFASFFVL**	Consensus (comb.lib./smm/nn)	0.01	1.1363	No	Positive	0.2194
	HLA-DPA1^∗^01 : 03/DPB1^∗^02 : 01	**NISIYFFASFFVLYF**	Consensus (comb.lib./smm/nn)	0.01	1.5428	No	Positive	1
MSP1	HLA-DPA1^∗^03 : 01/DPB1^∗^04 : 02	FLGISFLLILMLILY	Consensus (comb.lib./smm/nn)	0.01	1.3457	No	Negative	64
	HLA-DPA1^∗^03 : 01/DPB1^∗^04 : 02	NFLGISFLLILMLIL	Consensus (comb.lib./smm/nn)	0.01	1.1366	No	Negative	60
	HLA-DPA1^∗^03 : 01/DPB1^∗^04 : 02	LGISFLLILMLILYS	Consensus (comb.lib./smm/nn)	0.02	0.9541	No	Negative	54
	HLA-DPA1^∗^03 : 01/DPB1^∗^04 : 02	GISFLLILMLILYSF	Consensus (comb.lib./smm/nn)	0.03	0.8777	No	Negative	48
	HLA-DPA1^∗^03 : 01/DPB1^∗^04 : 02	ISFLLILMLILYSFI	Consensus (comb.lib./smm/nn)	0.03	1.6407	No	Negative	46
MSP3	HLA-DRB3^∗^02 : 02	HLYIYINNVASKEIV	NetMHCIIpan	0.01	0.2830	Yes	Positive	4
	HLA-DRB3^∗^02 : 02	LHLYIYINNVASKEI	NetMHCIIpan	0.01	0.6264	Yes	Negative	4
	HLA-DRB3^∗^02 : 02	LYIYINNVASKEIVK	NetMHCIIpan	0.01	0.1614	Yes	Positive	4
	HLA-DRB3^∗^02 : 02	YIYINNVASKEIVKK	NetMHCIIpan	0.02	-0.2207	Yes	Positive	1
	HLA-DRB3^∗^02 : 02	LLHLYIYINNVASKE	NetMHCIIpan	0.09	0.6712	Yes	Negative	-0.3417
MSP4	HLA-DRB1^∗^01 : 01	CVELLSLASSSLNLI	Consensus (comb.lib./smm/nn)	0.1	-0.0621	No	Positive	1
	HLA-DRB1^∗^01 : 01	ECVELLSLASSSLNL	Consensus (comb.lib./smm/nn)	0.1	0.1481	Yes	Positive	1
	HLA-DRB1^∗^01 : 01	VELLSLASSSLNLIF	Consensus (comb.lib./smm/nn)	0.1	0.0252	Yes	Positive	1
	HLA-DRB1^∗^01 : 01	IECVELLSLASSSLN	Consensus (comb.lib./smm/nn)	0.16	0.2052	Yes	Positive	1
	HLA-DPA1^∗^01 : 03/DPB1^∗^02 : 01	LNLIFNSFITIFVVI	Consensus (comb.lib./smm/nn)	0.22	1.0058	No	Negative	8
MSP6	HLA-DPA1^∗^01 : 03/DPB1^∗^02 : 01	MNKIYNITFLFILLN	Consensus (comb.lib./smm/nn)	0.14	0.4987	No	Negative	11
	HLA-DPA1^∗^01 : 03/DPB1^∗^02 : 01	NKIYNITFLFILLNL	Consensus (comb.lib./smm/nn)	0.14	0.7500	No	Negative	13
	HLA-DRB1^∗^13 : 02	ILLNLYINENNFIRN	Consensus (smm/nn/sturniolo)	0.22	1.5255	No	Negative	-0.2297
	HLA-DRB1^∗^13 : 02	LLNLYINENNFIRNE	Consensus (smm/nn/sturniolo)	0.23	1.0849	Yes	Negative	-0.2188
	HLA-DRB1^∗^13 : 02	LNLYINENNFIRNEL	Consensus (smm/nn/sturniolo)	0.27	1.2208	Yes	Negative	-0.4038
Ralp1	LA-DRB3^∗^02 : 02	NKKFKLNESMKNHFY	NetMHCIIpan	0.04	0.7270	No	Negative	-0.5034
	HLA-DPA1^∗^01 : 03/DPB1^∗^02 : 01	FIIVYCFISSFYLIK	Consensus (comb.lib./smm/nn)	0.06	0.7382	Yes	Negative	4
	LA-DRB3^∗^02 : 02	KKFKLNESMKNHFYN	NetMHCIIpan	0.06	0.4953	No	Negative	-0.5117
	HLA-DPA1^∗^01 : 03/DPB1^∗^02 : 01	IIVYCFISSFYLIKS	Consensus (comb.lib./smm/nn)	0.07	0.5982	Yes	Negative	1
	HLA-DPA1^∗^01 : 03/DPB1^∗^02 : 01	FFIIVYCFISSFYLI	Consensus (comb.lib./smm/nn)	0.08	1.1158	Yes	Negative	6
RESA	HLA-DPA1^∗^01 : 03/DPB1^∗^02 : 01	IVTLLRFFFEKRLSM	Consensus (comb.lib./smm/nn)	0.24	0.7305	No	Negative	-0.1734
	HLA-DRB1^∗^07 : 01	ESLRWIFKHVAKTHL	Consensus (comb.lib./smm/nn)	0.28	-0.2477	No	Negative	-0.1925
	HLA-DRB1^∗^07 : 01	LRWIFKHVAKTHLKK	Consensus (comb.lib./smm/nn)	0.28	0.2922	Yes	Negative	-0.1144
	HLA-DRB1^∗^07 : 01	RWIFKHVAKTHLKKS	Consensus (comb.lib./smm/nn)	0.28	0.3480	Yes	Negative	-0.2612
	HLA-DRB1^∗^07 : 01	SLRWIFKHVAKTHLK	Consensus (comb.lib./smm/nn)	0.28	0.0077	Yes	Negative	-0.3372
Rh5	HLA-DRB3^∗^01 : 01	NDDSYRYDISEEIDD	Consensus (comb.lib./smm/nn)	0.06	0.5481	Yes	Positive	0.1695
	HLA-DRB3^∗^01 : 01	**DDSYRYDISEEIDDK**	Consensus (comb.lib./smm/nn)	0.09	0.6564	No	Positive	0.1793
	HLA-DRB3^∗^01 : 01	DSYRYDISEEIDDKS	Consensus (comb.lib./smm/nn)	0.11	0.7195	Yes	Positive	0.1783
	HLA-DRB3^∗^01 : 01	KMGSYIYIDTIKFIH	Consensus (comb.lib./smm/nn)	0.11	0.3265	No	Negative	-0.3428
	HLA-DRB3^∗^01 : 01	KNDDSYRYDISEEID	Consensus (comb.lib./smm/nn)	0.11	0.3230	No	Negative	-0.3932
Ripr	HLA-DRB1^∗^01 : 01	**CQGMYISLRSVHVHT**	Consensus (comb.lib./smm/nn)	0.1	0.4897	No	Positive	0.2683
	HLA-DRB1^∗^01 : 01	**GMYISLRSVHVHTHN**	Consensus (comb.lib./smm/nn)	0.1	0.6017	No	Positive	0.1345
	HLA-DRB1^∗^01 : 01	NCQGMYISLRSVHVH	Consensus (comb.lib./smm/nn)	0.1	0.4426	No	Negative	-0.1838
	HLA-DRB1^∗^01 : 01	**QGMYISLRSVHVHTH**	Consensus (comb.lib./smm/nn)	0.1	0.7164	No	Positive	0.0831
	HLA-DRB4^∗^01 : 01	IFFTLLIIILIKKTS	Consensus (comb.lib./smm/nn)	0.12	1.1012	No	Negative	116
Ron2	HLA-DRB1^∗^01 : 01	GTKSFYSLPTILTAN	Consensus (comb.lib./smm/nn)	0.01	-0.5149	No	Negative	-0.8120
	HLA-DRB1^∗^01 : 01	KSFYSLPTILTANSD	Consensus (comb.lib./smm/nn)	0.01	-0.0287	No	Negative	-0.4571
	HLA-DRB3^∗^02 : 02	**LVRGNYIGNINNIAR**	NetMHCIIpan	0.01	0.8144	No	Positive	0.4925
	HLA-DRB3^∗^02 : 02	RGNYIGNINNIARND	NetMHCIIpan	0.01	0.8171	Yes	Positive	0.2910
	HLA-DRB1^∗^01 : 01	TGTKSFYSLPTILTA	Consensus (comb.lib./smm/nn)	0.01	-0.6603	No	Negative	-0.8987
Sera5	HLA-DQA1^∗^01 : 01/DQB1^∗^05 : 01	KSYISLFFILCVIFN	Consensus (comb.lib./smm/nn)	0.11	1.0472	No	Negative	-0.086
	HLA-DQA1^∗^01 : 01/DQB1^∗^05 : 01	**MKSYISLFFILCVIF**	Consensus (comb.lib./smm/nn)	0.11	1.1703	No	Positive	0.1021
	HLA-DRB1^∗^11 : 01	MDIFNNLKRLLIYHS	Consensus (smm/nn/sturniolo)	0.12	-0.1486	No	Negative	1
	HLA-DQA1^∗^01 : 01/DQB1^∗^05 : 01	**SYISLFFILCVIFNK**	Consensus (comb.lib./smm/nn)	0.12	0.7201	No	**Positive**	0.050
	HLA-DRB1^∗^11 : 01	EMDIFNNLKRLLIYH	Consensus (smm/nn/sturniolo)	0.18	-0.1137	No	Negative	-0.2916
Sera8	HLA-DRB1^∗^07 : 01	TLYQLKRVHSNMLIN	Consensus (comb.lib./smm/nn)	0.03	-0.0784	Yes	Negative	-0.0028
	HLA-DRB1^∗^07 : 01	VTLYQLKRVHSNMLI	Consensus (comb.lib./smm/nn)	0.03	-0.0198	Yes	Positive	0.1241
	HLA-DRB1^∗^07 : 01	LYQLKRVHSNMLINC	Consensus (comb.lib./smm/nn)	0.06	0.2755	Yes	Negative	1
	HLA-DRB1^∗^07 : 01	LVTLYQLKRVHSNML	Consensus (comb.lib./smm/nn)	0.07	-0.1590	Yes	Positive	0.1447
	HLA-DRB1^∗^07 : 01	YQLKRVHSNMLINCF	Consensus (comb.lib./smm/nn)	0.09	-0.3701	No	Negative	1

**Table 7:** Regional and global population coverage analysis of predicted HTL epitopes in 14 selected erythrocytic stage *P. falciparum* antigens.

Area	Class II MHC allele coverage		
	Coverage	Average hit^a^	pc90^b^
Central Africa	99.21%	13.28	5.74
East Africa	99.17%	13.87	5.74
West Africa	99.43%	12.0	5.0
North Africa	72.9%	5.14	0.37
South Africa	7.65%	0.25	0.11
Central America	77.45%	4.23	0.44
North America	99.98%	17.77	11.7
South America	98.51%	13.54	5.51
West Indies	78.33%	5.49	0.46
Europe	99.83%	17.37	11.43
East Asia	76.66%	5.09	0.43
Northeast Asia	93.635	11.45	2.47
South Asia	98.92%	15.51	8.4
Southeast Asia	69.41%	3.08	0.33
Southwest Asia	70.01%	4.07	0.33
Oceania	93.69%	11.03	2.63
World	97.17%	14.21	4.57
Average	84.23	9.88	3.86

^a^Average number of epitope hits/HLA combinations recognized by the population. ^b^Minimum number of epitope hits/HLA combinations recognized by 90% of the population.

## Data Availability

The data used to support the findings of this study are available from the corresponding author upon request.

## Supplementary Materials

Supplementary Materials Supplementary Figure 1: comparative in silico simulation of elicited antibody titers, based on AMA1, CyRPA, Rh5, MSP1, and Sera5 proteins, using C-ImmSim web server. Supplementary Figure 2: comparative *in silico* simulation of elicited cytokines, based on AMA1, CyRPA, Rh5, MSP1, and Sera5 proteins, using C-ImmSim web server. Supplementary Figure 3: comparative *in silico* simulation of T helper cell population state, based on AMA1, CyRPA, Rh5, MSP1, and Sera5 proteins, using C-ImmSim web server.
