# Supplementary material for: Computational Clues of Immunogenic Hotspots in Plasmodium falciparum Erythrocytic Stage Vaccine Candidate Antigens: In Silico Approach
Source: Biomed Res Int. 2022 Oct 13;2022:5886687. doi: 10.1155/2022/5886687 (PMC9584662; doi:10.1155/2022/5886687)

**Supplementary Figure 1.** Comparative *in silico* simulation of elicited antibody titers, based on AMA1, CyRPA, Rh5, MSP1 and Sera5 proteins, using C-ImmSim web server.


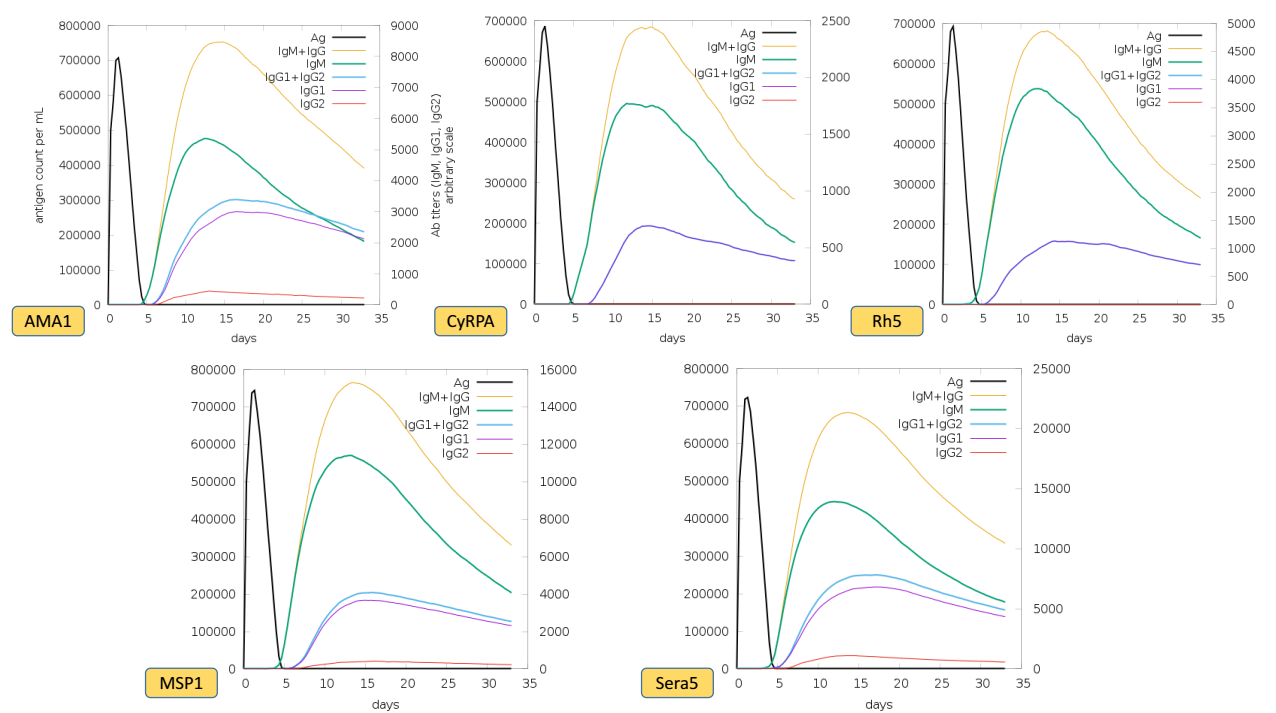


**Supplementary Figure 2.** Comparative *in silico* simulation of elicited cytokines, based on AMA1, CyRPA, Rh5, MSP1 and Sera5 proteins, using C-ImmSim web server.


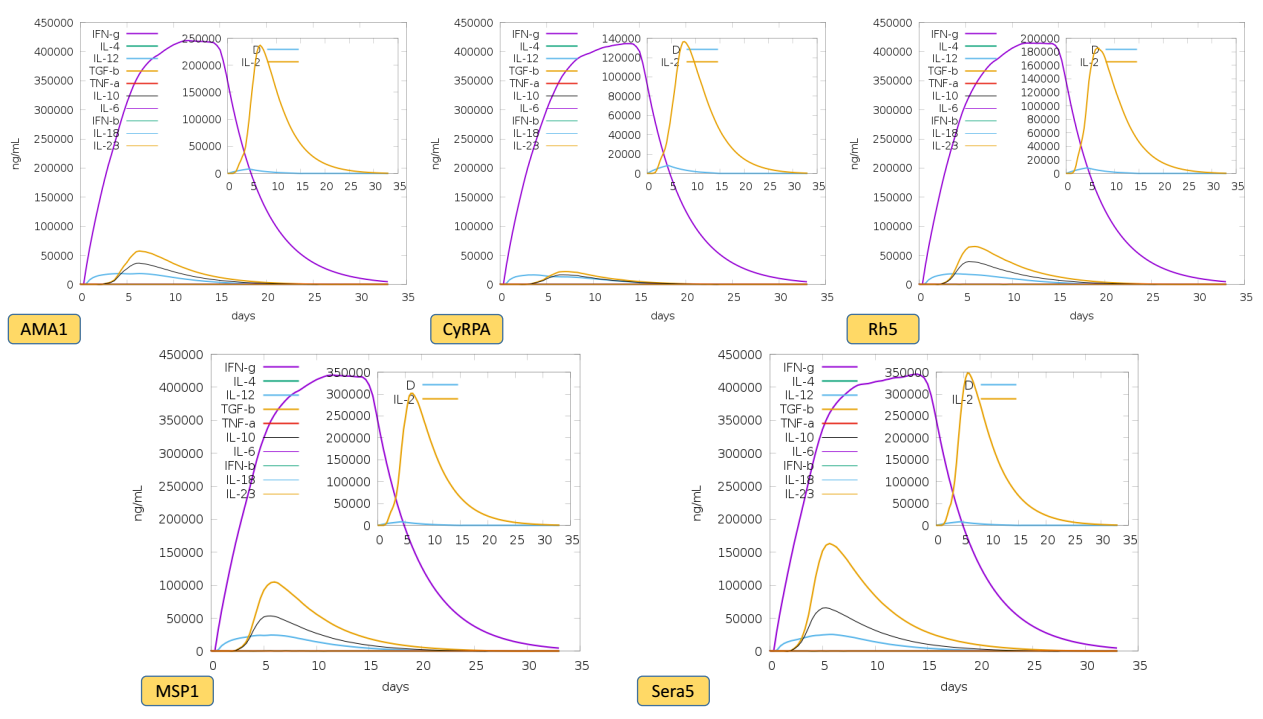


**Supplementary Figure 3.** Comparative *in silico* simulation of T helper cell population state, based on AMA1, CyRPA, Rh5, MSP1 and Sera5 proteins, using C-ImmSim web server.


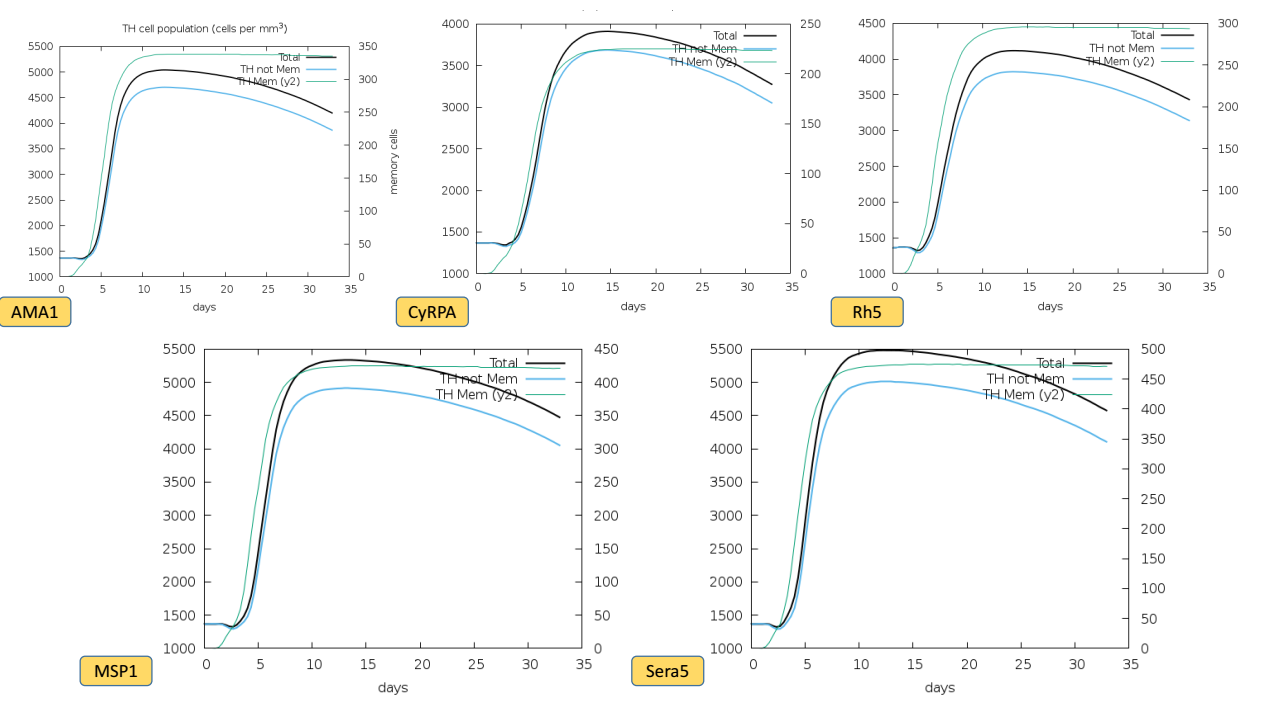

Supplement: Supplementary Materials — Supplementary Figure 1: comparative in silico simulation of elicited antibody titers, based on AMA1, CyRPA, Rh5, MSP1, and Sera5 proteins, using C-ImmSim web server. Supplementary Figure 2: comparative in silico simulation of elicited cytokines, based on AMA1, CyRPA, Rh5, MSP1, and Sera5 proteins, using C-ImmSim web server. Supplementary Figure 3: comparative in silico simulation of T helper cell population state, based on AMA1, CyRPA, Rh5, MSP1, and Sera5 proteins, using C-ImmSim web server. [file 5886687.f1.docx]
